# Effect of sprayable, highly adhesive hydrophobized gelatin microparticles on esophageal stenosis after endoscopic submucosal dissection: an experimental study in a swine model

**DOI:** 10.1007/s10388-024-01090-8

**Published:** 2024-10-15

**Authors:** Hiroki Yano, Fumisato Sasaki, Hidehito Maeda, Shohei Uehara, Masayuki Kabayama, Yusuke Fujino, Akihito Tanaka, Makoto Hinokuchi, Shiho Arima, Shinichi Hashimoto, Shuji Kanmura, Shima Ito, Akihiro Nishiguchi, Tetsushi Taguchi, Akio Ido

**Affiliations:** 1 Digestive and Lifestyle Diseases, Kagoshima University Graduate School of Medical and Dental Sciences, 8-35-1, Sakuragaoka, Kagoshima, 890-8520 Japan; 2 Research Center for Macromolecules and Biomaterials, National Institute for Materials Science, Tsukuba, Japan; 3 Graduate School of Science and Technology, Degree Programs in Pure and Applied Sciences, University of Tsukuba, Tsukuba, Japan

**Keywords:** Endoscopic submucosal dissection, Esophageal stricture, Inflammation, Gelatin, Swine

## Abstract

**Background:**

Esophageal mucosal resection for superficial esophageal cancer can lead to postoperative esophageal stricture, with current preventive measures being insufficient. Sprayable wound dressings containing hydrophobized microparticles exhibit strong adhesion. This study aimed to investigate the preventive effects of hydrophobized microparticles on esophageal stenosis following endoscopic submucosal dissection.

**Methods:**

Circumferential esophageal endoscopic submucosal dissection was performed on miniature swine (n = 6). Swine were categorized into two groups: those sprayed with hydrophobized microparticles (sprayed group) and those not sprayed (non-sprayed group). Hydrophobized microparticles were sprayed onto the sprayed group on Days 0, 3, and 7 of endoscopic submucosal dissection. The non-sprayed group underwent endoscopy on the same days. Esophageal stricture rate, submucosal inflammatory cell infiltration, submucosal fibrosis, and thickening of the muscular layer were compared between the groups on Day 14 of endoscopic submucosal dissection.

**Results:**

Spraying of hydrophobized microparticles was easily performed using an existing endoscopic spraying device. The esophageal stricture rate was significantly lower in the sprayed group than in the non-sprayed group (76.1% versus 90.6%, p < 0.05). The sprayed group showed suppression of inflammatory cell infiltration in the submucosal layer (p < 0.01) and thickening of the muscular layer (p < 0.01).

**Conclusions:**

Sprayable tissue-adhesive hydrophobized microparticles reduce the stricture rate after esophageal ESD by inhibiting inflammatory cell infiltration, submucosal fibrosis, and thickening of the muscular layer. The use of hydrophobized microparticles for preventing post-endoscopic submucosal dissection esophageal stenosis offers a promising avenue for clinical applications in endoscopic procedures, potentially improving patient outcomes.

**Supplementary Information:**

The online version contains supplementary material available at 10.1007/s10388-024-01090-8.

## Introduction

Endoscopic submucosal dissection (ESD) is a well-established and widely adopted technique for endoscopic resection, increasingly utilized worldwide [1]. ESD has enabled resection of extensive lesions; however, several adverse events have been reported, with esophageal stenosis being the most common [1].

Extensive esophageal mucosal resection for superficial esophageal cancer often results in postoperative esophageal stricture, significantly decreasing patients’ quality of life [2]. Such postoperative stricture is particularly frequent in cases of extensive dissection of approximately ≥ 75% of the circumference, with the incidence of postoperative strictures for circumferential lesions possibly reaching 100% [3, 4]. Guidelines for esophageal cancer published in Japan in 2022 strongly recommend preventive measures following endoscopic treatment of esophageal cancer [5]. However, despite various stenosis-preventive measures, preventive measures for patients undergoing full circumferential resection are still insufficient.

In our previous study, a hexanoyl (Hx:C6) group-modified alkaline-treated gelatin porous film (HAG) induced proper healing by decreasing submucosal fibrosis in post-ESD gastric ulcers in miniature swine [6, 7]. Thereafter, we developed a sprayable wound dressing comprising multifunctional hydrophobized microparticles (hMPs) derived from swine gelatin. This dressing showed strong tissue adhesion in wet environments and effectively suppressed submucosal fibrosis in post-ESD gastric ulcers in miniature swine [8]. Furthermore, we successfully developed a sprayable wound dressing made with fine particles of pollock gelatin [9]. We have previously also reported the strong adhesive effect of hMPs in terms of closing gastrointestinal perforation models [9, 10]. We have also demonstrated an anti-inflammatory effect on post-ESD ulcers in a duodenal ESD animal model [10].

We hypothesized that highly adhesive hMPs would effectively prevent strictures after total esophageal ESD by tightly adhering to post-ESD ulcers and suppressing inflammation and fibrosis in the submucosal and muscle layers. Here, we aimed to investigate the efficacy of hMPs in preventing stenosis after esophageal ESD.

## Materials and methods

### Experimental animals

Six miniature swine (male or female, 6 months old; 11–15 kg; Kagoshima Miniature Swine Research Center, Kagoshima, Japan) were used in this study. Premedication for these miniature swine involved intramuscular injections of 15 mg/kg ketamine (Daiichi Sankyo Propharma, Tokyo, Japan) and 2 mg/kg xylazine (Bayer Yakuhin, Osaka, Japan), followed by sevoflurane inhalation anesthesia (Maruishi Pharmaceutical, Osaka, Japan). An endotracheal tube (100/199/065; Smith Medical Japan, Tokyo, Japan) was inserted, and anesthesia was maintained using sevoflurane. The animals were allowed free access to water on the day of ESD and solid food the following day.

### Ethical statement

All experiments were approved by the Animal Care and Use Committee of Kagoshima University (approval number: MD 20015). Animal care, housing, and surgery were performed in accordance with the guidelines and regulations of the Committee for Animal Research at Kagoshima University, Japan.

### hMPs

**Fig. 1:**
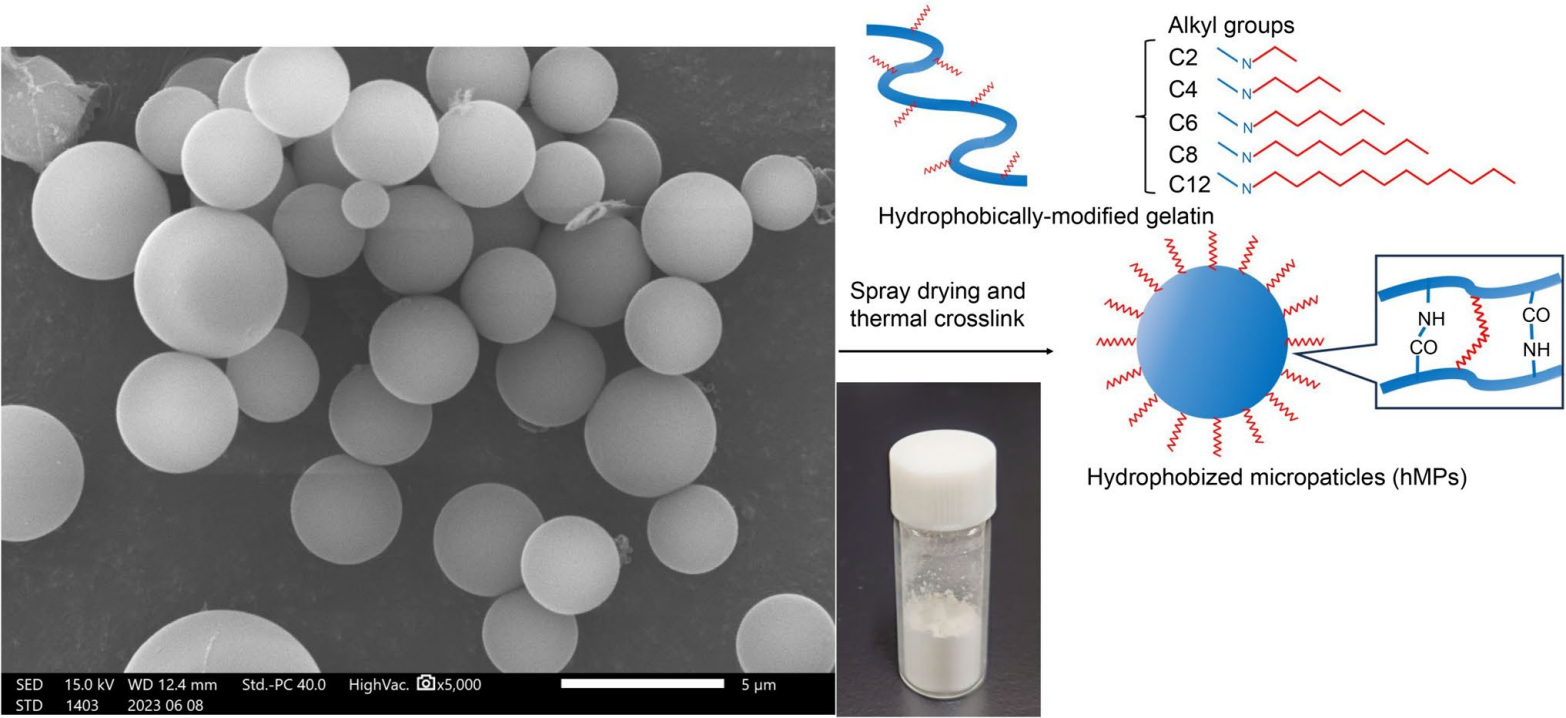
Scanning electron microscopy image of hMPs. In this study, hydrophobically modified Alaska ollock gelatin is prepared by reductive amination of gelatin and aldehyde. hMPs are prepared using a coacervation method in a water/ethanol mixed solvent. *hMPs* hydrophobized microparticles

Hydrophobically modified Alaska pollock gelatin (hm-ApGltn) was prepared by reductive amination between gelatin and aldehyde [11]. hMPs were prepared using a coacervation method in a water/ethanol mixed solvent [12]. The optimized alkyl chain length (decyl groups, C10) and degree of substitution (43% with amino groups in ApGltn) of the hydrophobic groups improved the mechanical strength of the hydrogel formed by hydration and fusion of the microparticles. Scanning electron microscopy confirmed that the obtained hMPs possessed micrometer-sized particles (Fig. 1).

### ESD procedure

**Fig. 2:**
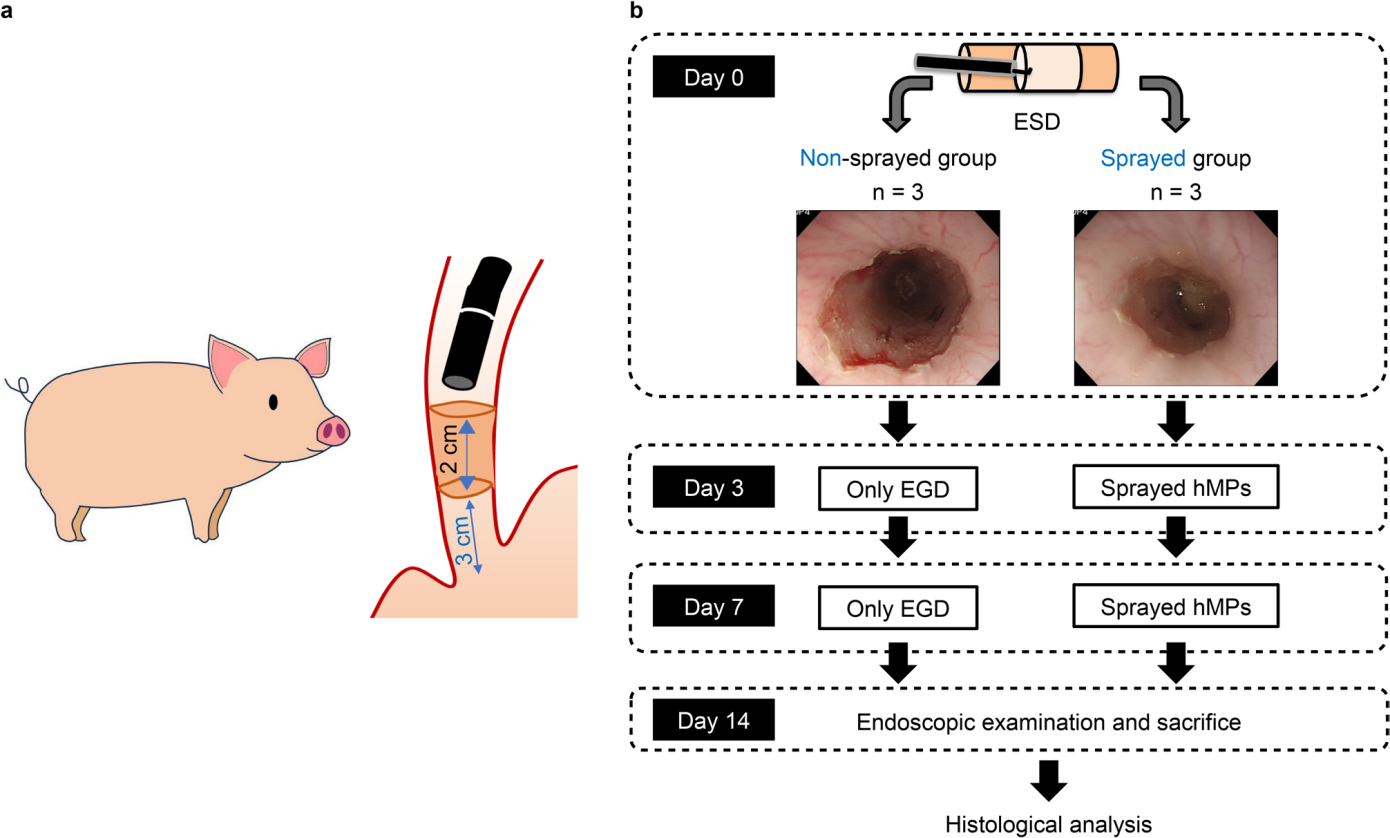
ESD procedure and grouping in this study. **a** A full circumferential artificial ulcer with a 20-mm-long diameter is created at the lower esophagus (3 cm mouth side from the EG junction) using the ESD technique. **b** The swine are divided into two groups: the sprayed and non-sprayed. The animals in the sprayed group are sprayed with hMPs on Days 0, 3, and 7 of ESD. The animals in the non-sprayed group underwent endoscopy on the same days. *hMPs* hydrophobized microparticles, *ESD* endoscopic submucosal dissection, *EG junction* esophagogastric junction

We established an ESD-induced esophageal stricture model. A full circumferential artificial ulcer with a 20-mm-long diameter was created at the lower esophagus [3 cm from the esophagogastric (EG) junction to the mouth] using the ESD technique. Artificial esophageal ulcers were created by an expert endoscopist who performed ESD using an upper gastrointestinal endoscope (GIF-Q260J; Olympus, Tokyo, Japan) and video scope system (EVIS LUCERA CV-260SL; Olympus). A glycerin solution containing small amounts of indigo carmine (glycerol injection; Hikari Pharmaceutical, Tokyo, Japan) was injected into the submucosa. The mucosa and submucosal layers were incised using an electric knife (Hook Knife J, KD-625LR; Olympus) and an electrosurgical generator (Pulse Cut Fast mode, 30 W, ESG-100; Olympus) (Fig. 2a). The diameter of the ulcer was measured using forceps (M2-K4; Olympus).

### Delivery of hMPs

hMPs (200 mg) were packed into small vials. A battery-powered Alto shooter endoscopic injector (Alto Shooter Kaigen, Tokyo, Japan), which can be used to apply powdered drugs, was used to spray the hMPs. A small vial of hMPs was attached directly to the Alto shooter, and the nozzle was removed from the endoscope duct and sprayed on the ulcer (Online Resource 1). The anterior, posterior, right, and left walls of circumferentially resected esophageal ulcers were sprayed. Thus, 800 mg of hMPs (four vials) were used per ulcer.

### Study schedule

The swine were divided into the sprayed and non-sprayed groups, and hMPs (800 mg/ulcer) were sprayed on the sprayed group on Days 0, 3, and 7 of ESD. The non-sprayed group underwent endoscopy on the same days. In a previous study on HAG developed before hMPs [6], HAG remained on the ulcer surface on day 3 after gastric ESD; however, on day 7, it did not remain on the ulcer surface but in the granulation tissue. Based on these results, we performed hMPs spraying on days 3 and 7 after esophageal ESD in this study with the expectation of residual and higher efficacy. In this study, the animals were carefully observed for vomiting. If vomiting occurred, the swine were offered non-solid foods. All efforts were made to minimize animal suffering. All miniature swine were sacrificed on Day 14 of ESD by intravenous administration of a lethal dose of sodium pentobarbital (Kyoritsu Seiyaku Co., Ltd., Tokyo, Japan). A histological evaluation was performed. (Fig. 2b).

### Evaluation of esophageal stricture rate

Because it was difficult to quantitatively analyze an esophageal stricture using endoscopic video images, each stricture was evaluated on the basis of the stricture rates of the resected sample after fixation. The resected esophageal specimen was pasted and fixed, and the stenosis rate was derived [13, 14]. Specifically, stricture rate was evaluated as the ratio of the diameter of the most stenotic tubular structure to that of the normal area located 2 cm toward the mouth from the most stenotic area of the esophageal post-ESD ulcer. The diameters of the normal and most stenotic areas were measured using ImageJ version 1.52u (National Institutes of Health, Bethesda, MD, USA).

### Histological analyses

Specimens were fixed in 10% neutral-buffered formalin (Kenei Pharmaceutical, Osaka, Japan) for 48 h, and each lesion was sliced at 4-mm intervals. The slices were then embedded in a paraffin block, cut into 2-μm-thick sections, and stained with hematoxylin and eosin (H&E) or Azan. Inflammatory cells infiltrating the submucosal layers in 12 random fields of view (400 × magnification) were counted in the H&E-stained tissues under a microscope. Damage to the muscularis propria was evaluated using Azan staining. The thickness of the muscularis propria was quantified on the basis of the ratio of the muscularis propria thickness in the ulcerated part (U) to that of the non-ulcerated (NU) part (U/NU) [6]

### Immunohistochemistry

Immunohistochemical staining was performed to evaluate fibrosis in the submucosa.

Fibrosis was determined using anti-tissue inhibitor of metalloproteinases-1 (TIMP-1) antibody diluted 1:8000 (AbCam, Cambridge, MA, USA), anti-matrix metalloproteinase-2 (MMP-2) antibody diluted 1:1000 (AbCam), and anti-matrix metalloproteinase-9 (MMP-9) antibody diluted 1:500 (AbCam). Similar to the counting of inflammatory cells, positively expressing cells infiltrating the submucosa were counted in 12 randomly selected fields of view (400 × magnification). To compare the balance between tissue inhibitor of metalloproteinases (TIMPs) and metalloproteinases (MMPs), the ratio was calculated by dividing the number of TIMP-1-positive-expressing cells by the number of MMP-2- and MMP-9-positive-expressing cells.

### Statistical analyses

The statistical significance of differences between the two groups was calculated using the Student’s *t* test or Mann–Whitney U test, depending on the results of the Kolmogorov–Smirnov test for normality. Statistical significance was set at p < 0.05. All statistical analyses were performed using EZR (version 1.54; Saitama Medical Center, Jichi Medical University, Saitama, Japan) [15] and a graphical user interface for R (version 2.13.0; R Foundation for Statistical Computing, Vienna, Austria).

## Results

### hMPs can reduce the esophageal stricture rate

**Fig. 3:**
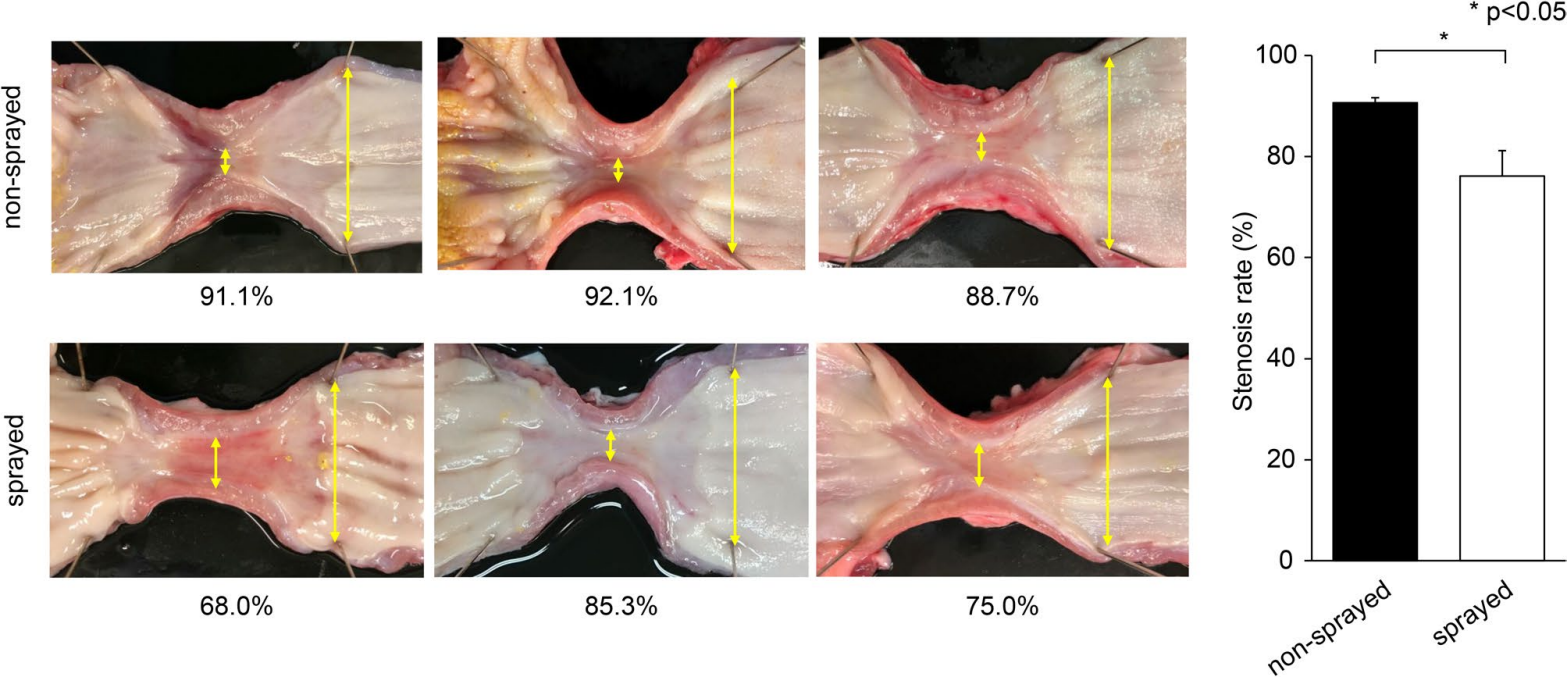
hMPs can significantly reduce esophageal stricture rate in full circumferential esophageal ESD. After sacrificing on Day 14, the esophageal stricture rate is compared on the basis of macroscopic findings. The esophageal stricture rate is significantly lower in the sprayed group than in the non-sprayed group (76.11% ± 5.03% vs. 90.65% ± 1.00%, respectively; p < 0.05). *hMPs* hydrophobized microparticles, *ESD* endoscopic submucosal dissection

ESD was performed safely in all swine without any adverse events. The esophagus of all swine was observed endoscopically on Days 3, 7, and 14 of ESD. The endoscope could not pass through the stenosis in either group because of severe esophageal stricture. Endoscopically, the strictures were more severe in the non-sprayed group than in the sprayed group (Online Resource 2). To accurately evaluate the stenosis rate, the esophageal stenosis rate was compared with macroscopic findings after sacrifice on Day 14. The esophageal stricture rate was significantly lower in the sprayed group than in the non-sprayed group (76.11% ± 5.03% vs. 90.65% ± 1.00%, respectively; p < 0.05) (Fig. 3).

### Histological evaluation

**Fig. 4:**
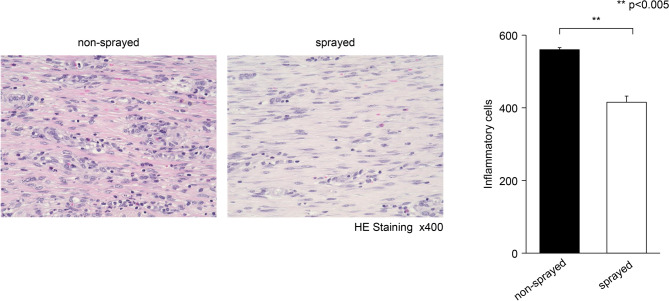
hMPs can decrease inflammatory cell infiltration. Histological observation of the submucosal tissues after 14 days shows that the number of invaded inflammatory cells is lower in the tissues sprayed with hMPs than in the non-sprayed tissue (415.36 ± 16.76 /HPF vs. 559.97 ± 5.50 /HPF, respectively; p < 0.005). *hMPs* hydrophobized microparticles

**Fig. 5:**
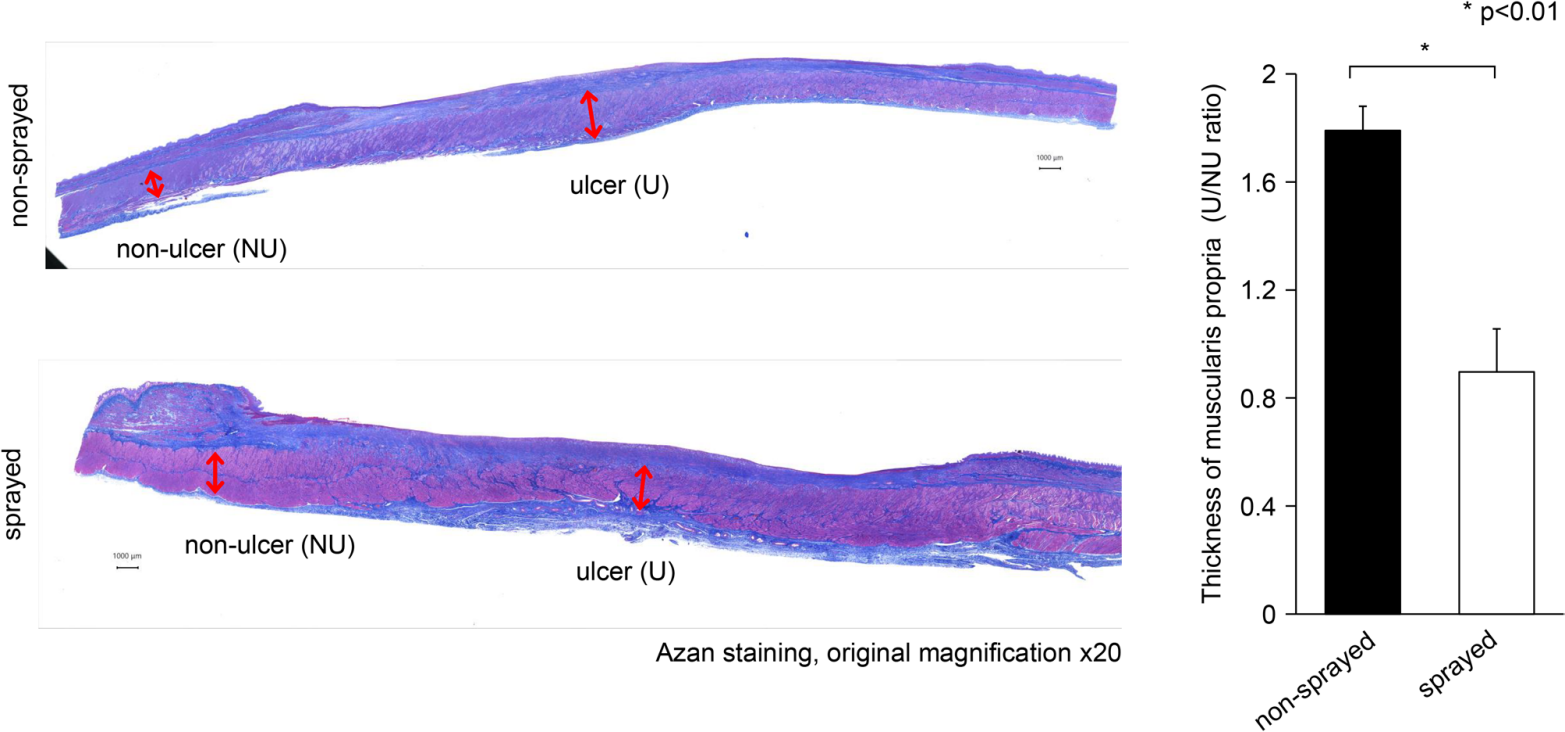
hMPs can suppress the thickening of the muscular layer. Thickening of the muscular layer is significantly suppressed in the sprayed group compared with that in the non-sprayed group (U/NU ratio, 0.90 ± 0.16 vs. 1.79 ± 0.09, respectively; p < 0.01). *hMPs* hydrophobized microparticles, *HE* hematoxylin and eosin

**Fig. 6:**
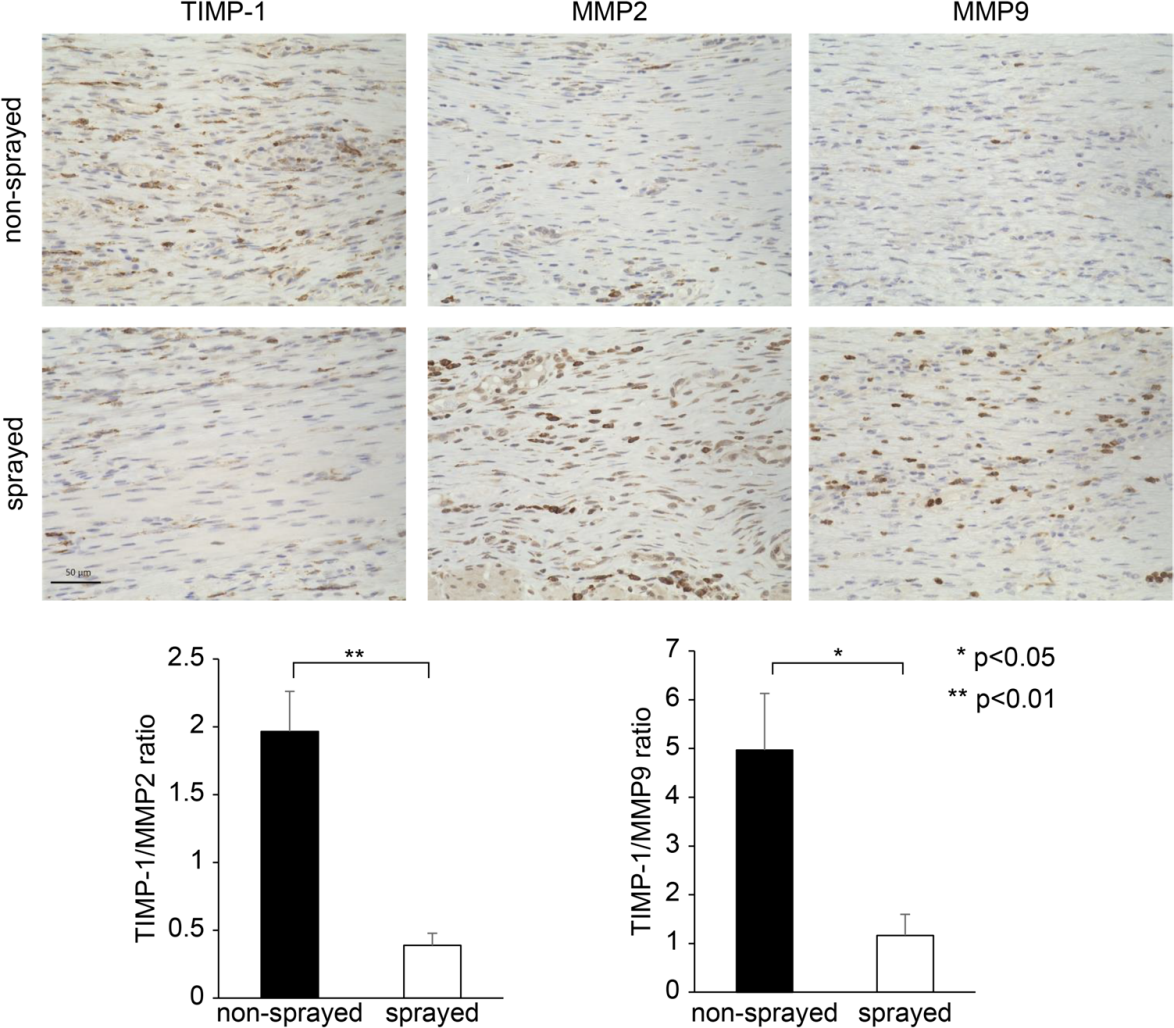
hMPs can decrease the ratio of TIMP-1 to MMP-2 positive cells (TIMP-1/MMP-2 ratio) and TIMP-1 to MMP-9 positive cells (TIMP-1/MMP-9 ratio). The TIMP-1/MMP-2 ratio in the submucosal layer is significantly lower in the sprayed group than in the non-sprayed group (0.39 ± 0.09 vs. 1.97 ± 0.30, respectively; p < 0.01). Similarly, the TIMP-1/MMP-9 ratio in the submucosal layer is significantly lower in the sprayed group (1.17 ± 0.43 vs. 4.97 ± 1.16, respectively; p < 0.05). *hMPs* hydrophobized microparticles, *MMP* matrix metalloproteinase, *TIMP-1* tissue inhibitor of metalloproteinases-1

Histological observation of the submucosal tissues after 14 days of ESD showed that the number of invaded inflammatory cells was lower in the tissues in the sprayed group than in the non-sprayed group (415.36 ± 16.76 /HPF vs. 559.97 ± 5.50 /HPF, respectively; p < 0.01) (Fig. 4). Thickening of the muscular layer was significantly suppressed in the sprayed group compared with that in the non-sprayed group (U/NU ratio, 0.90 ± 0.16 vs. 1.79 ± 0.09, respectively; p < 0.01) (Fig. 5). Additionally, the ratio of TIMP-1- to MMP-2-positive cells in the submucosal layer (TIMP-1/MMP-2 ratio) was significantly lower in the sprayed group than in the non-sprayed group (0.39 ± 0.09 vs. 1.97 ± 0.30, respectively; p < 0.01). Similarly, the ratio of TIMP-1- to MMP-9-positive cells in the submucosal layer (TIMP-1/MMP-9 ratio) was significantly lower in the sprayed group than in the non-sprayed group (1.17 ± 0.43 vs. 4.97 ± 1.16, respectively; p < 0.05) (Fig. 6).

## Discussion

**Fig. 7:**
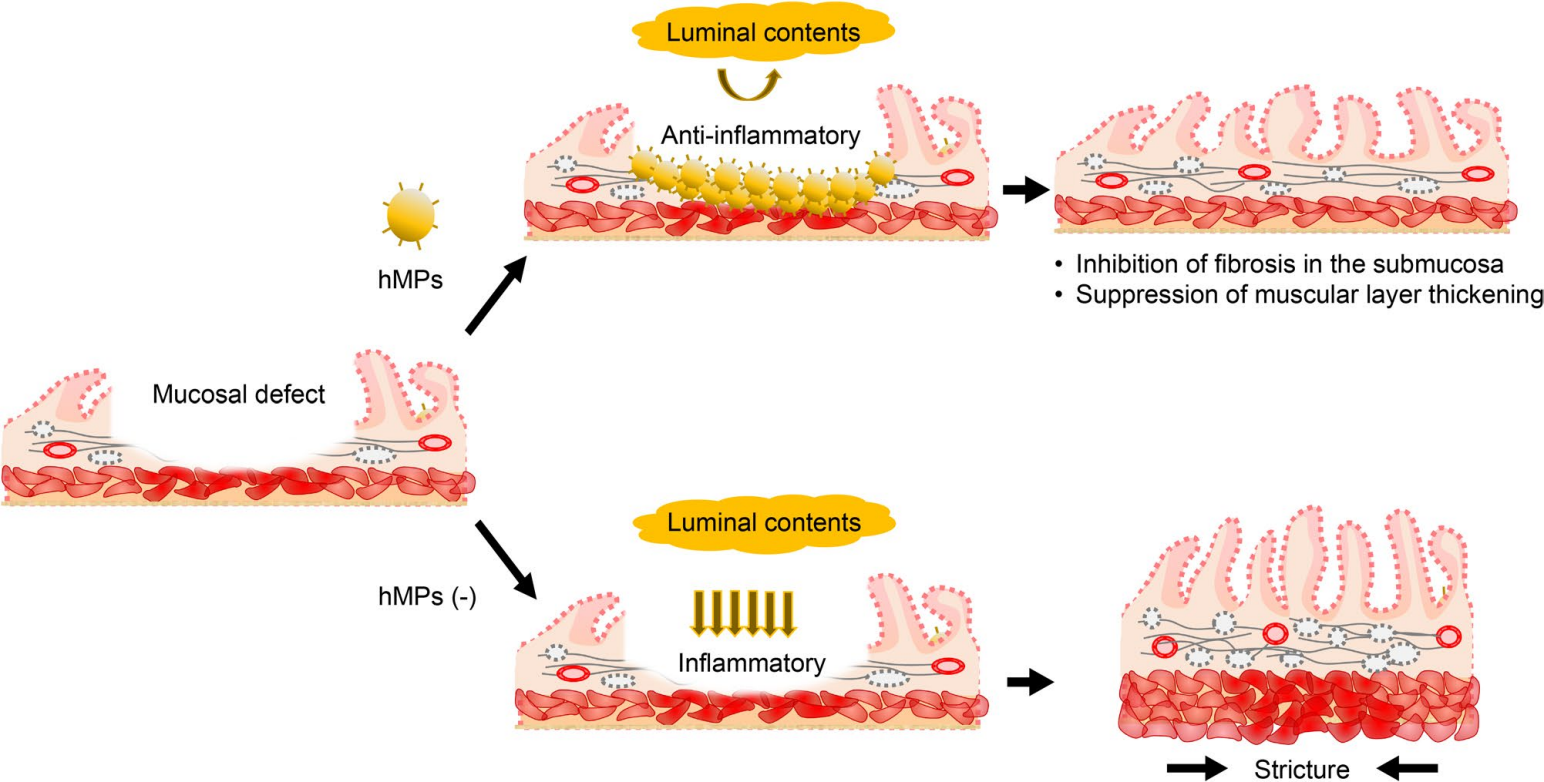
Sprayable, tissue-adhesive hMPs may serve as a promising medical intervention to prevent post-esophageal ESD stricture. Our findings show that hMPs can efficiently suppress fibrosis in submucosal tissues via physical and biochemical interactions and prevent post-esophageal ESD stricture. *hMPs* hydrophobized microparticles, *ESD* endoscopic submucosal dissection

We showed that hMPs can efficiently suppress inflammation, inhibit fibrosis in submucosal tissues, and suppress the thickening of the muscular layer via physical and biochemical interactions after ESD. Consequently, we succeeded in showing that hMPs reduced the stricture rate after esophageal ESD (Fig. 7).

During the wound-healing process, such as in the case of ESD ulcers, inflammatory cells migrate to the ulcer area and form granulation tissues. Subsequently, fibroblasts, which are inflammatory cells, differentiate into myofibroblasts that contract ulcers. Fibroblasts are also known to produce collagen, contributing to fibrosis and the formation of hard scars [16].

The application of hMPs is believed to act as a barrier, preventing external stimuli and food from reaching the ulcer base; this barrier effect may lead to a reduction in the number of inflammatory cells, including fibroblasts, migrating to the ulcer site. Consequently, by inhibiting collagen production, ulcer contraction and fibrosis are suppressed. hMPs have the potential to decrease excess scarring through physical and biochemical interactions after ESD [10]. The present results indicate that hMPs may be useful in preventing stricture after esophageal ESD by reducing damage to the muscularis propria and suppressing the thickening of the muscular layer (Fig. 5).

Various methods have been employed to prevent postoperative stricture; however, their effectiveness is limited [17, 18]. In this regard, technologies for stricture prevention after ESD of esophageal neoplasias are emerging [19]. Collagen patches [14], autologous skin epidermal cell sheets [13], stomach mucosa transplantation [20], amniotic membrane grafts [21], and metal stents treated with extracellular matrix (ECM) and autologous platelet-rich plasma [22] are useful postoperative stricture mitigation methods. However, their clinical usefulness for post-ESD esophageal stenosis is unclear. We previously reported that HAG induces proper healing by decreasing inflammation in post-ESD gastric ulcers in miniature swine [6, 7]. Although HAG has strong self-adhesiveness, an anti-inflammatory effect, and an anti-fibrotic effect, its delivery to the esophagus via an endoscope is difficult. Therefore, we developed a sprayable wound dressing comprising hMPs made of swine gelatin that showed strong tissue adhesion in wet environments and effectively suppressed inflammation in rat skin ulcers and post-ESD gastric ulcers in miniature swine [8]. Ito et al. developed a sprayable wound dressing comprising hMPs derived from Alaska pollock gelatin [9]. The advantages of hMPs over other methods for preventing stenosis include ease of use and straightforward delivery to the ulcer site. This study utilized a spray formulation for the hMPs dosage form. In a previous study [6], a sheet formulation was employed. However, in the esophagus, which has a narrow lumen, applying the sheet to the optimal site was challenging due to its adhesive properties. Therefore, the sheet formulation was replaced with a spray formulation. Previously, we found that the anti-inflammatory effects of hMPs made of Alaska pollock gelatin, which gels quickly in swine models, were similar to those of hMPs derived from swine gelatin [10]. Here, the anti-inflammatory effects of hMPs made of Alaska pollock gelatin were also demonstrated in esophageal ulcers in miniature swine (Fig. 4).

In this study, the use of hMPs decreased the TIMP-1/MMP-2 and TIMP-1/MMP-9 ratios (Fig. 6). TIMP-1 and MMP are fibrosis-related proteins. To the best of our knowledge, no studies have reported on TIMP-1 expression in esophageal tissues; however, it is believed that an increase in TIMP-1 expression suppresses MMP activity in liver tissues, resulting in collagen deposition and the promotion of liver fibrosis [23]. In healthy liver tissues, a balance between TIMPs and MMPs controls the removal and assembly of the ECM [24]. During the wound-healing process, an imbalance between TIMPs and MMPs during the remodeling phase leads to excessive fibrosis of ECM components and promotes the formation of permanent fibrotic scars [25]. Erlewyn-Lajeunesse et al. examined the processes involved in airway remodeling during wheezing by measuring the TIMP-1-to-MMP-9 ratio [26]. MMP-9 and MMP-2 are gelatinases of the MMP family that degrade type IV collagen and gelatin substrates [27, 28]. TIMP-1 is a natural suppressor of MMP-9 and MMP-2 [29]. Previous studies have used a combination of MMP-2 and MMP-9 as MMPs suppressed by TIMP-1 [30], and we performed immunohistochemical staining for these MMPs in our study. TIMP-1 expression was suppressed compared with MMP-9 and MMP-2 expressions in the sprayed group. A TIMP-1/MMPs ratio that favors MMPs may prevent fibrosis due to ECM deposition. In other words, hMPs spraying is believed to maintain the balance between TIMP-1 and MMPs, resulting in ulcer healing with reduced fibrosis and stricture prevention by inhibiting excessive contraction.

Steroids, currently widely used to prevent stricture after esophageal ESD, are believed to prevent esophageal stricture by suppressing inflammation and inhibiting fibroblast proliferation and collagen formation, thereby reducing scarring [31]. Both steroids and hMPs share the common feature of suppressing inflammation, fibroblast proliferation, and collagen formation in the submucosal to muscular layers. We plan to conduct future studies using a combination of steroids and hMPs.

This study has some limitations. First, it was conducted using a small number of animals. The use of miniature swine, which are medium-to-large animals, made it challenging to utilize a larger sample size for this experiment. Second, the long-term effect of hMPs on post-ESD ulcers was not monitored. Further studies are required to examine the long-term anti-fibrotic and stenosis-preventive effects of hMPs on the submucosa. Third, a very strong stricture in occurred during full circumferential esophageal ESD in this miniature swine model. In this study, the endoscope could not pass through the esophageal stricture in a miniature swine full circumferential ESD, because it was unexpectedly severe. Post-ESD stricture in miniature pigs may be more severe than in humans; however, completely preventing esophageal stricture with hMP alone may be difficult. We plan to investigate the efficacy of hMPs in partial esophageal ESD and in combination with steroids. Fourth, there is a lack of studies addressing the safety of hMPs in humans. However, hMPs stimulate macrophages to produce MMP2, which promotes the degradation of hMPs to peptides. Gelatin itself is not immunogenic, and its degradation by MMP2 to peptides is considered safe [32]. Fifth, we had not been able to examine in detail how long hMPs remain in the tissues after spraying. Although we should have evaluated the persistence and dosage of hMPs and determined the administration schedule, we could not conduct multiple studies, because we used medium-sized animals (miniature pigs) in this study. In this study, all miniature swine were sacrificed at day 14 post-ESD, and no hMPs remained in the histological findings at that time. In conclusion, our results suggest that hMPs reduce the stricture rate after esophageal ESD by inhibiting inflammatory cell infiltration, submucosal fibrosis, and thickening of the muscular layer. With further studies in animal models and clinical trials in humans, hMPs may prove beneficial in clinical practice for preventing postoperative strictures after esophageal ESD.

## Supplementary Information

Below is the link to the electronic supplementary material.

Supplementary file1 (MP4 52307 kb)

Supplementary file2 (DOCX 164 kb)

## Data Availability

The data that support the findings of this study are available from the corresponding author, F.S., upon reasonable request.
