# Supplementary material for: Effect of sprayable, highly adhesive hydrophobized gelatin microparticles on esophageal stenosis after endoscopic submucosal dissection: an experimental study in a swine model
Source: Esophagus. 2024 Oct 15;22(1):95–104. doi: 10.1007/s10388-024-01090-8 (PMC11717788; doi:10.1007/s10388-024-01090-8)
Supplement: Supplementary file 2 — Supplementary file2 (DOCX 164 kb) [file 10388_2024_1090_MOESM2_ESM.docx]

**Online Resource 1**

**Effect of sprayable, highly adhesive hydrophobized gelatin microparticles on esophageal stenosis after endoscopic submucosal dissection: an experimental study in a swine model**

**Journal Name:** Esophagus

Hiroki Yano, MD^1^, Fumisato Sasaki, MD, PhD^1^*, Hidehito Maeda, MD, PhD^1^, Shohei Uehara, MD^1^, Masayuki Kabayama, MD, PhD^1^, Yusuke Fujino, MD^1^, Akihito Tanaka, MD, PhD^1^, Makoto Hinokuchi, MD, PhD^1^, Shiho Arima, MD, PhD^1^, Shinichi Hashimoto, MD, PhD^1^, Shuji Kanmura, MD, PhD^1^, Shima Ito, PhD^2,3^, Akihiro Nishiguchi, PhD^2^, Tetsushi Taguchi, PhD^2,3^, Akio Ido, MD, PhD^1^

^1^Digestive and Lifestyle Diseases, Kagoshima University Graduate School of Medical and Dental Sciences, Kagoshima, Japan

^2^Research Center for Macromolecules and Biomaterials, National Institute for Materials Science, Tsukuba, Japan

^3^Graduate School of Science and Technology, Degree Programs in Pure and Applied Sciences, University of Tsukuba, Tsukuba, Japan

***Corresponding author**: bungohs@m2.kufm.kagoshima-u.ac.jp

**
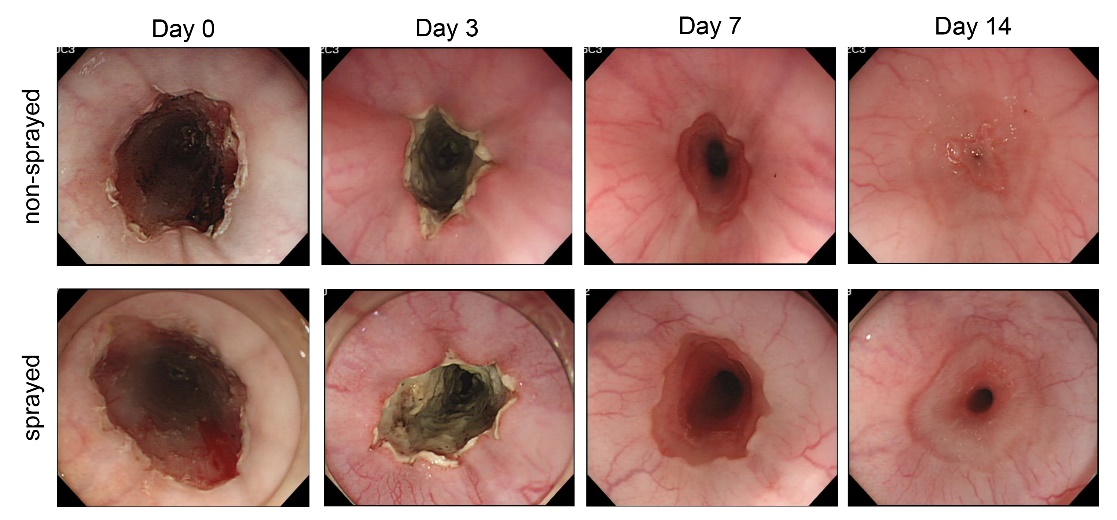
**

**Fig. S1 Temporal changes in endoscopic findings**

Endoscopic observations are performed on all miniature swine on the day of ESD and on Days 3, 7, and 14 of ESD. Severe strictures are observed in both groups. Stricture appears more severe in the non-sprayed group than in the sprayed group.

ESD: endoscopic submucosal dissection
